# Impaired cognitive function and decreased monoamine neurotransmitters in the *DNAJC12* gene knockout mouse model

**DOI:** 10.1186/s13023-025-03580-z

**Published:** 2025-02-08

**Authors:** Shunan Wang, Ming Shen, Bo Pang, Bo Zhou, Yuan Yuan, Mei Lu, Xiangling Deng, Min Yang, Shufang Liu, Qiong Wang, Mei Xue, Qisheng Xia, Zhixin Zhang

**Affiliations:** 1 Graduate School, Beijing University of Chinese Medicine, Beijing, China; 2 International Medical Services, China-Japan Friendship Hospital, Beijing, China; 3 Department of Pediatrics, China-Japan Friendship Hospital, Beijing, China; 4 Child Healthcare Center, Children’s Hospital, Capital Institute of Pediatrics, Beijing, China; 5 Department of Pediatrics, Women and Children’s Hospital, School of Medicine, Xiamen University, Fujian, China; 6 Institute of Clinical Medical Sciences, China-Japan Friendship Hospital, Beijing, China

**Keywords:** *DNAJC12* gene knockout mice, Hyperphenylalanine, CRISPR/Cas9, Aromatic amino acid hydroxylase, Morris water maze, Neurotransmitters

## Abstract

**Background:**

Hyperphenylalaninemia, a prevalent amino acid metabolism disorder, often results in cognitive impairment. Recent studies have identified a rare variant of this disorder caused by mutations in the *DNAJC12* gene. The specific mechanisms by which *DNAJC12* mutations lead to hyperphenylalaninemia and the lack of an animal model for study remain significant gaps in understanding.

**Purpose:**

This study aims to elucidate the role of DNAJC12 in intellectual disability and explore the mechanisms by which DNAJC12 deficiency leads to hyperphenylalaninemia through developing a *DNAJC12* gene knockout mouse model.

**Methods:**

We thoroughly examined the clinical features and genetic mutations evident in two patients with biallelic mutations in the *DNAJC12* gene. Based on the patient’s mutation locations, we determined the target site for the knockout utilizing CRISPR/Cas9 technology. To assess the impact of these mutations on *DNAJC12* expression, we used quantitative real-time PCR and Western blotting techniques to measure mRNA and protein levels, respectively. The Morris water maze test was administered to evaluate the cognitive functions of the mice. Additionally, we utilized High-Performance Liquid Chromatography (HPLC) to measure serum aromatic amino acids and brain monoamines, facilitating an investigation into the metabolism of phenylalanine and tyrosine.

**Results:**

We reported two patients with mutations in the *DNAJC12* gene. Case 1 carried the mutations c.158-1G > A and c.262 C > T in the *DNAJC12* gene. He presented with nocturnal eye closure, crying, and arching back in reverse tension before treatment, suggesting a neurotransmitter metabolism disorder. Case 2 carried the mutations c.473 C > G, and c.102 deletion in the *DNAJC12* gene. He showed elevated blood phenylalanine levels, although further clinical details were not available. Based on the patients’ mutation locations, exons 2–4 of the *DNAJC12* gene were targeted and eliminated. In our animal model experiments, *DNAJC12* gene knockout mice exhibited a complete absence of *DNAJC12* expression at both mRNA and protein levels. These knockout mice showed significant deficits in learning and memory performance as assessed by the Morris water maze test. Quantitative real-time PCR analysis indicated no significant differences in the levels of aromatic amino hydroxylases between knockout and wild-type mice. However, Western blot analysis revealed a substantial reduction in phenylalanine hydroxylase (PAH) protein levels in the liver of knockout mice, while tyrosine hydroxylase (TH) and tryptophan hydroxylase 2 (TPH2) expression remained unchanged. HPLC analysis demonstrated increased serum Phe concentrations and decreased levels of brain neurotransmitters in the knockout group.

**Conclusions:**

We report two patients with four novel *DNAJC12* mutations (c.158-1G > A, c.262 C > T, c.473 C > G, c.102delT), expanding the mutation spectrum. Based on the patients’ mutation location, we established the first *DNAJC12* gene knockout mouse model. The knockout mice exhibit hyperphenylalaninemia, impaired cognitive function, and decreased monoamine neurotransmitters. DNAJC12 deficiency may contribute to the clinical phenotype via the PAH pathway, potentially at the post-transcriptional level.

## Introduction

Hyperphenylalaninemia (HPA), the most prevalent inborn error of amino acid metabolism, traditionally encompasses phenylalanine hydroxylase (PAH) deficiency and tetrahydrobiopterin (BH4) deficiency [1]. Recently, a rare form caused by *DNAJC12* gene mutations has been recognized. Yair Anikster et al. [2] first reported cases of HPA due to *DNAJC12* gene mutations, followed by numerous subsequent reports [3–12].The phenotypic spectrum of *DNAJC12* mutation varies widely, ranging from intellectual disability and severe neurological symptoms to milder or even normal phenotypes. Most patients with BH4 loading test (20 mg/kg/day) show decreased serum Phe concentrations. However, these patients exhibit normal urine pterins and DHPR activity. Although medication treatment (oral BH4 or neurotransmitter precursors such as L-Dopa/carbidopa and 5-hydroxytryptophan) or a low-phenylalanine diet can partially alleviate symptoms, they cannot entirely prevent them. Consequently, understanding the mechanisms by which *DNAJC12* gene mutations cause clinical symptoms and finding effective treatments is crucial.

The *DNAJC12* gene encodes the DNAJC12 protein, a type III member of the HSP40/DNAJ protein family [13]. DNAJC proteins, functioning as molecular chaperones for the HSP70 family, assist in the proper folding and intracellular stability of their client proteins [13].DNAJC12 is hypothesized to be a co-chaperone for aromatic amino acid hydroxylases (AAAHs), which include PAH, tyrosine hydroxylase (TH), and tryptophan hydroxylase (TPH). These enzymes, utilizing BH4 and divalent iron atoms as co-factors, catalyze essential amino acid conversions [14]. AAAHs use tetrahydrobiopterin (BH4) and divalent iron atoms as co-factors to catalyze the conversion of phenylalanine to tyrosine, tyrosine to L-dopa, and tryptophan to 5-hydroxytryptophan respectively. Huttlin EL et al. have shown DNAJC12’s interactions with various proteins, including TH, TPH1, and TPH2 [15]. Anikster et al. further confirmed these interactions and observed reduced PAH activity in fibroblasts with a specific homozygous missense variant compared to control fibroblasts [2]. Despite being recognized as a specific co-chaperone of AAAHs, inconsistencies in research exist. For example, Gallego et al. noted that *DNAJC12* gene variants may affect PAH and TH protein expression, but not TPH2^7^. To elucidate the role of DNAJC12 in intellectual disability and explore the mechanisms by which DNAJC12 defficiency leads to hyperphenylalaninemia, we have constructed the first *DNAJC12* gene knockout mouse model and conducted corresponding experiments.

## Methods

### Patient clinical features and ethics statement

All clinical data were obtained with parental consent and approved by the China-Japan Friendship Hospital Ethics Committee. We investigated two patients exhibiting hyperphenylalaninemia with biallelic mutations in the *DNAJC12* gene. The patients were initially identified through newborn screening and finally diagnosed via genetic testing. Next-generation sequencing was utilized for genetic testing, followed by Sanger sequencing to confirm mutation sites. These mutations were compared against the 1000 Genomes Project, gnomAD ALL (http://gnomad-sg.org/), and ExAC databases (http://exac.broadinstitute.org/). Variants were classified following the guidelines established by the American College of Medical Genetics and Genomics (ACMG).

### Preparation of DNAJC12 gene knockout mouse model

*DNAJC12*-/- mice were generated using CRISPR/Cas9 technology. All mice were C57BL/6J background in this study. The procedures for animal experiments were approved by the China-Japan Friendship Hospital Animal Care Welfare Committee.

**Targeting Strategy**: The murine *DNAJC12* gene included 5 exons. The target site for knockout was selected based on the patient’s mutation locations. Specific sgRNA and ssDNA were designed and synthesized based on these target sites. sgRNA1 (matching forward strand of gene): AGAACTATATTAAGGGTCGTAGG; sgRNA2 (matching forward strand of gene): CCGGAGATATCAAAGATCGTGGG; sgRNA3 (matching forward strand of gene): GGTCGTAGGCAGCATTAGTGGGG; sgRNA4 (matching forward strand of gene): AGATGGACTTCGAACTCATAGGG.

#### Micromanipulation and fertilized egg transfer

Cas9 mRNA, sgRNA, and oligo donor were co-injected into fertilized C57BL/6J eggs. Subsequently, the embryos were transplanted into the fallopian tubes of recipient female mice. The resulting offspring constituted the F0 generation.

**PCR screening and Expanded Breeding of F0 Generation Mice**: PCR screening was conducted to identify the positive mice. PCR Primers 1: F1: 5’-TTGGGCACTAGGCACTGACCTTGA-3’; R1: 5’-ACCTCTCATGCTTAATAGTCATCCTCTC-3’.PCR Primers 2: F1: 5’-TTGGGCACTAGGCACTGACCTTGA-3’; R2: 5-TCAATGGATGGATGTGTAGCAGT-3’. Mice were positive(homozygotes or heterozygotes) if a band was amplified by primer1 and wild-type if a band was amplified by primer2. Positive F0 mice were crossbred with wild-type counterparts to produce the genetically stable F1 generation. Further, the inbreeding of F1 mice led to the birth of the F2 generation.

**Sequencing Confirmation**: *DNAJC12* knockout was confirmed in F2 generation mice through PCR and gene sequencing. The PCR primers were the same as above. Sequencing Primer (R1): 5’ -ACCTCTCATGCTTAATAGTCATCCTCTC-3’.

### Morris water maze

The Morris Water Maze (MWM) test was performed to assess learning and memory abilities. Mice aged 6–8 weeks were used. The experimental setup consisted of a circular pool equipped with cameras and data analysis software (Beijing Yusheng Technology Development Ltd., Beijing, China). The pool was divided into four quadrants, with one quadrant containing a submerged platform. The water was maintained at 28 °C.

Cued Learning Test: On the first day, the cued learning test was initiated. The platform was set 1 cm above the water surface, and mice were released from various quadrants to locate it.

Navigation Test: From days 2 to 6, the navigation test was carried out. The mice were trained to find the platform submerged below the water surface. They were released from four different starting positions, with each trial lasting up to 90 s. A latency of 90 s was recorded if a mouse failed to find the platform within this time. Metrics such as trajectory, speed, latency, and distance traveled were recorded.

Probe Test: On the seventh day, a probe test was performed. The platform was removed, and each mouse was released twice from random starting positions, with each trial again lasting 90 s.

### Real-time PCR

The primer sequences in the PCR reaction were the following. The primers were obtained from Tsingke Biotechnology Co. Ltd., Beijing, China. GAPDH: F- GGTTGTCTCCTGCGACTTCA; R-TGGTCCAGGGTTTCTTACTCC. DNAJC12: F-ATTGGGCTGTCAGAAGTAAGAA; R-TGCTTCATTTTCTCTGGGTTTG. PAH: F-CGAGACAAGGAAAAGAACACAG; R-GCTCCATAGCTGAGAATCTGAT. TH: F-GTTTCAGTGCACACAGTACATC; R-CACCGTGGAGAGTTTTTCAATT. TPH2: F-GAGCAGCAAGACAGCGGTAGTG; R-TTCGTCGGGACCTCCTGGATTC.

### Western blot

After the Morris water maze, the mice were sacrificed. Their brain and liver were stripped quickly and put into liquid nitrogen. Western blot analysis was conducted to detect the expression of DNAJC12 protein in brain and liver tissues, PAH in liver tissues, TH in striatum tissues, and TPH2 in raphe nucleus tissues.

### High-pressure liquid chromatography (HPLC)

Serum phenylalanine (Phe), tyrosine(Tyr), and brain monoamines were measured by HPLC Analysis (Sykam chromatography, Munich, Germany). The corpus striatum was stripped for dopamine (DA), dihydroxyphenyl acetic acid (DOPAC), homovanillic acid (HVA), and the raphe nuclei for 5- hydroxytryptamine (5-HT), 5-hydroxyindoleacetic acid (5-HIAA).

### Statistical analysis

Data were analyzed in STATA SE/14.1. Figures were completed by GraphPad Prism 8.0.1. For normally distributed data, a *t*-test was used to compare the groups, and data are presented as mean ± standard deviation; Otherwise, the Mann-Whitney U test would be conducted, and data are presented as median and interquqrtile range. Statistical significance is indicated as ^*^P < 0.05, ^**^P < 0.01, ^***^P < 0.001, ^****^P < 0.0001.

## Results

### Clinical features of two patients with DNAJC12 biallelic mutations

**Fig. 1:**
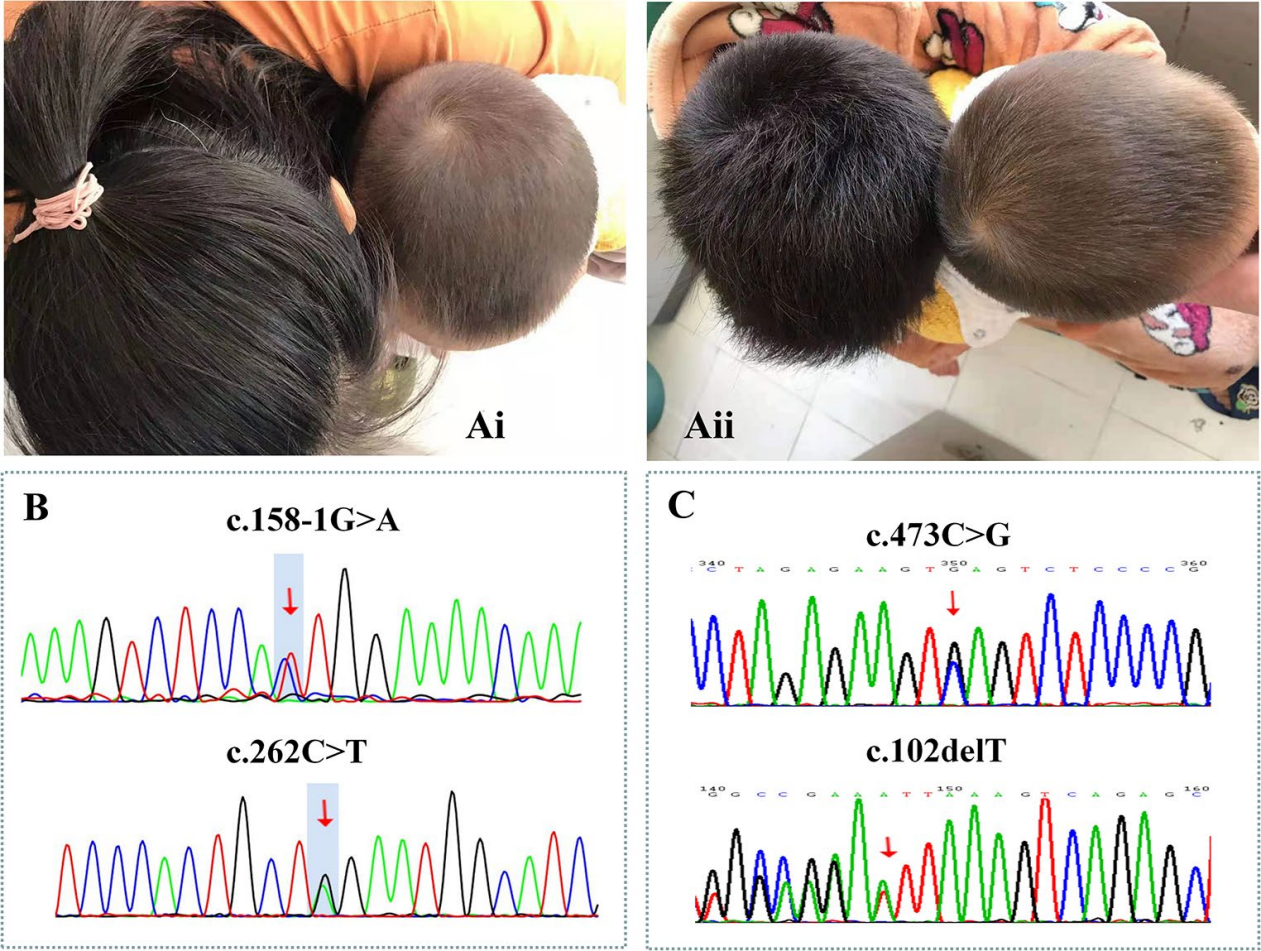
Clinical manifestations and mutations of patients with mutations in *DNAJC12* gene. (**A**)Comparison of the hair color of case 1 with his mother (left image) and with his brother (right image). (**B**)Case 1 had the heterozygous variants c.158-1G > A and c.262 C > T (p.Q88X). (**C**) Case 2 had the compound heterozygous variants c.473 C > G (p.S158X) and c.102delT (p.F34Lfs*33)

Case 1, a male, was born to non-consanguineous Han parents in Fujian Province. Neonatal screening revealed a Phe concentration of 305.47 µmol/L. On day 24, he commenced a low-phenylalanine diet, which reduced his blood Phe concentration to 30.4µmol/L by day 34, allowing him to resume a normal diet. Before 15 months of age, his Phe levels fluctuated between 131.99 and 291µmol/L, generally remaining below 240µmol/L until the age of 15 months. However, after 15 months, his Phe levels predominantly exceeded 240µmol/L, peaking up at 419.62 µmol/L. Clinically, the patient manifested with nocturnal eye closure, crying, and arching back in reverse tension. These symptoms resolved following treatment with Sapropterin dihydrochloride (1.25 mg/kg/day), L-dopa (1.04 mg/kg/day), and 5-hydroxytryptophan (0.83 mg/kg/day). At 1 year and 11 months, his height and weight fell within the normal range. A developmental assessment utilizing a diagnostic table designed for children aged 0 to 6 years revealed normal neural development. Additionally, this child exhibited yellow hair, whereas the rest of the family members had black hair (Fig. 1A). The change of hair color has been not reported in previous studies. Genetic analysis revealed a compound heterozygous mutation in the *DNAJC12* gene: c.158-1G > A and c.262 C > T (p.Q88X) (Fig. 1B). Both mutations are novel and were not reported in the 1000 Genomes Project, gnomAD ALL, or ExAC databases. Based on ACMG guidelines, these mutations were classified as pathogenic according to (PVS1 + PM2_supporting + PM3).

Case 2, a full-term male infant with a birth weight of 2800 g, was born to parents from Henan and Fujian provinces. Initial newborn screening indicated an elevated phenylalanine concentration of 171.6 µmol/L. Further investigations, including urinary pterin levels and dihydropyridine reductase (DHPR) activity tests, yielded normal results. Genetic testing identified a compound heterozygous mutation in the *DNAJC12* gene, consisting of c.473 C > G (p.S158X) and c.102delT (p.F34Lfs*33) (Fig. 1C). Both mutations were absent from the 1000 Genomes Project, gnomAD ALL, or ExAC databases. According to ACMG guidelines, these mutations were classified as pathogenic (PVS1 + PM2_supporting + PM3). Unfortunately, the patient was lost to follow-up, and further clinical details could not be obtained.

### Generation of DNAJC12 gene knockout mouse model

**Fig. 2:**
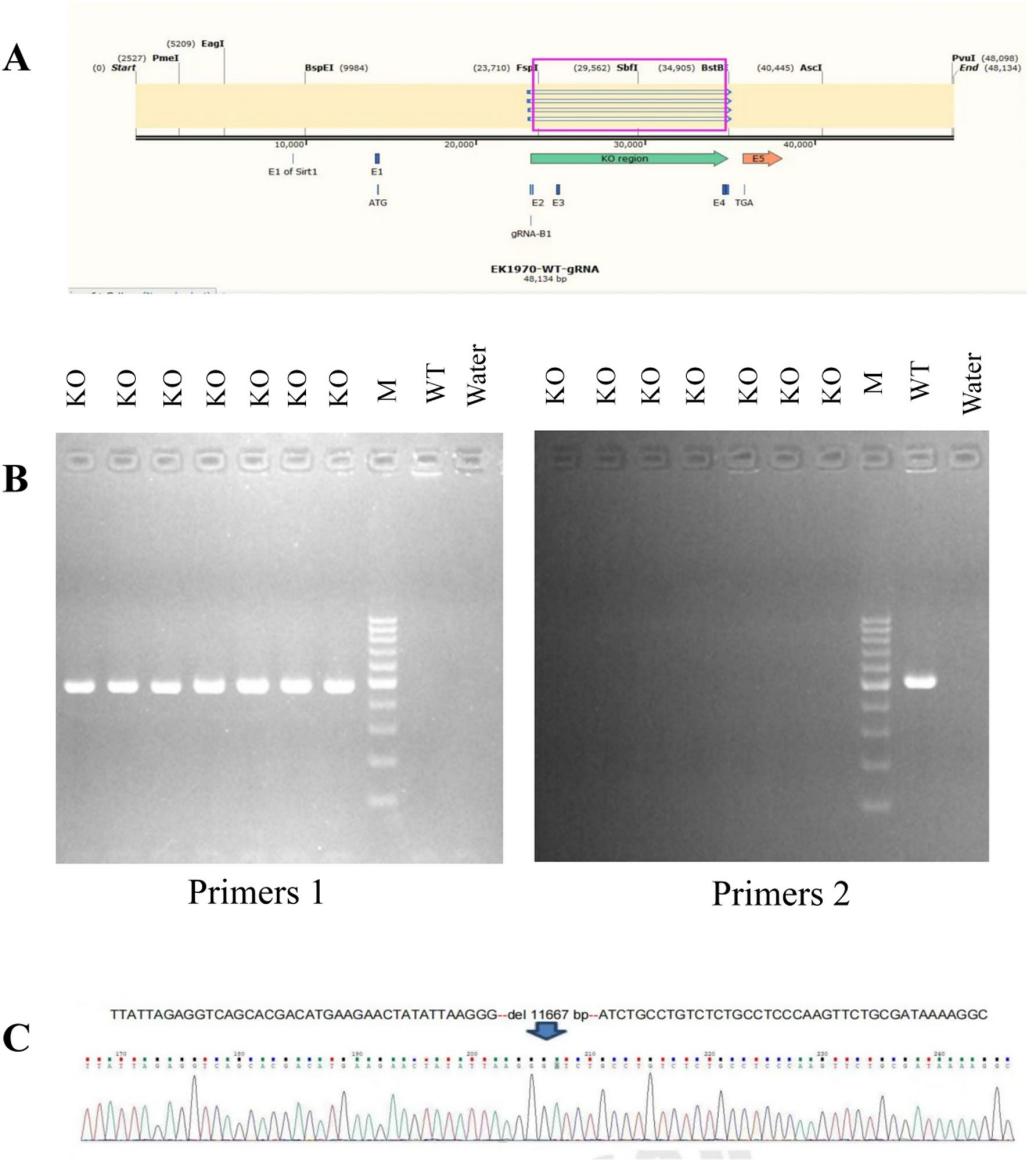
Generation of *DNAJC12* gene knockout mouse model. (**A**) Vector targeting DNAJC12 gene exon 2–4 was established with the primers of gRNA. (**B**) PCR screening: In the KO group, Primer1 amplified a band, and primer2 did not amplify a band. Wild-type group primer1 did not amplify a band, and primer2 amplified a band. (**C**) Gene sequencing: 11,667 bp in Exon 2–4 were deleted

**Fig. 3:**
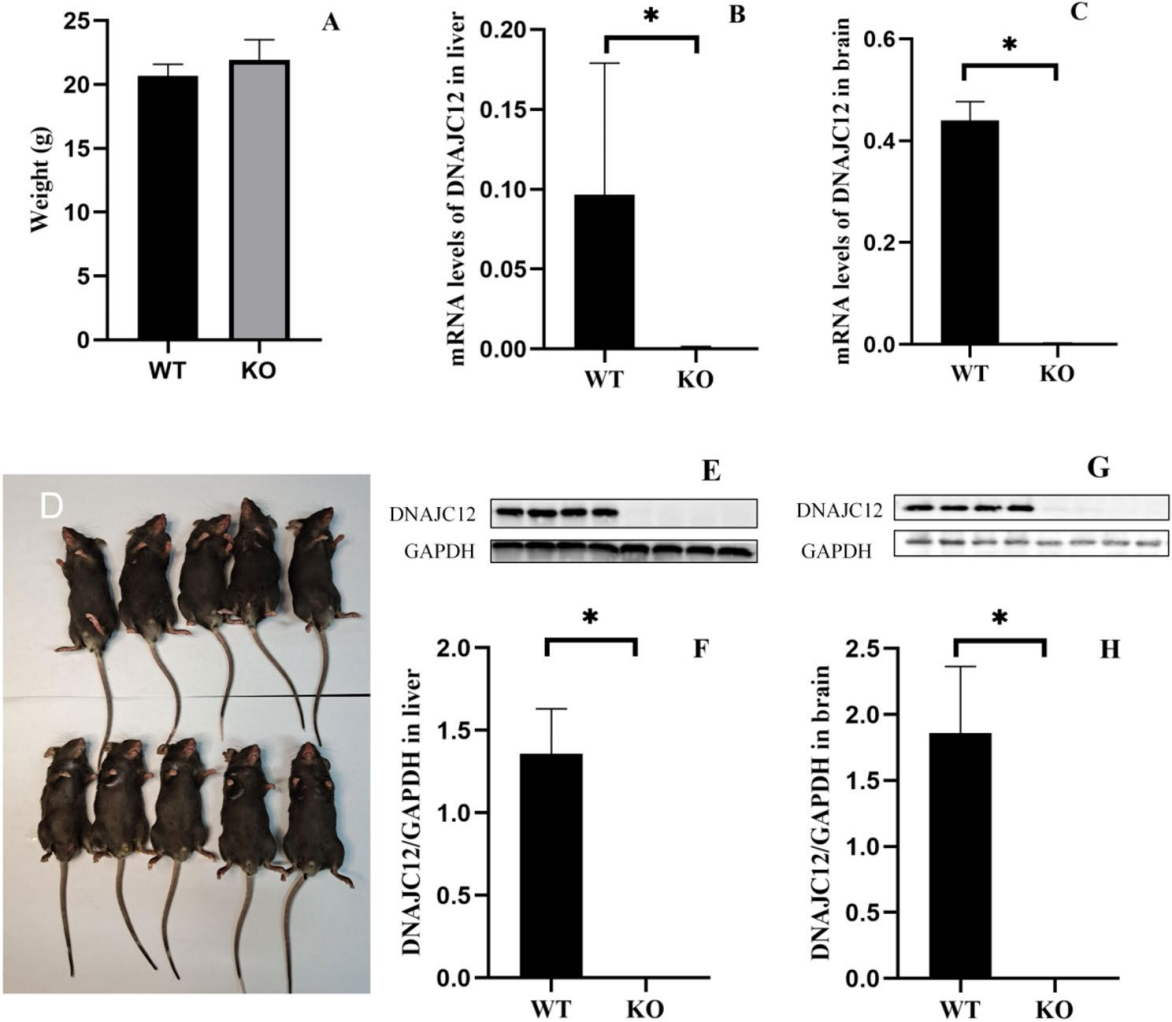
Characteristics of *DNAJC12* gene knockout mouse model. (**A**) Weight between WT mice (*n* = 12) and KO mice (*n* = 26). (**D**) Appearance of WT mice (the upper) and KO mice (the lower). (**B**, **C**, **E**, **F**, **G**, **H**) DNAJC12 expression in the liver and brain of WT mice and KO mice by RT-PCR (KO mice: *n* = 8; WT mice: *n* = 8) and WB (KO mice: *n* = 10; WT mice: *n* = 9). Data are presented as the mean ± SD. *P < 0.05. Unpaired t-tests were used to compare two groups of data

Given that all four mutation sites are situated on Exons 2 to 4, *DNAJC12* gene Exon 2–4 was targeted and knocked out. The mouse model was confirmed by PCR and Sanger sequencing (Fig. 2A, B and C). RT-PCR and Western blot analysis indicate DNAJC12 is not detected in the liver and brain in *DNAJC12* knockout mice (Fig. 3B, C, E, F, G and H), which confirmed the successful genetic modification of the *DNAJC12* gene. No significant difference in coat color, appearance, or weight (Fig. 3A and D) was observed between *DNAJC12*-/- mice and *DNAJC12*+/+ mice when they were 6 to 8 weeks old. In addition, no signs of dystonia or dyskinesia were observed in the model mice.

### Morris water maze

**Fig. 4:**
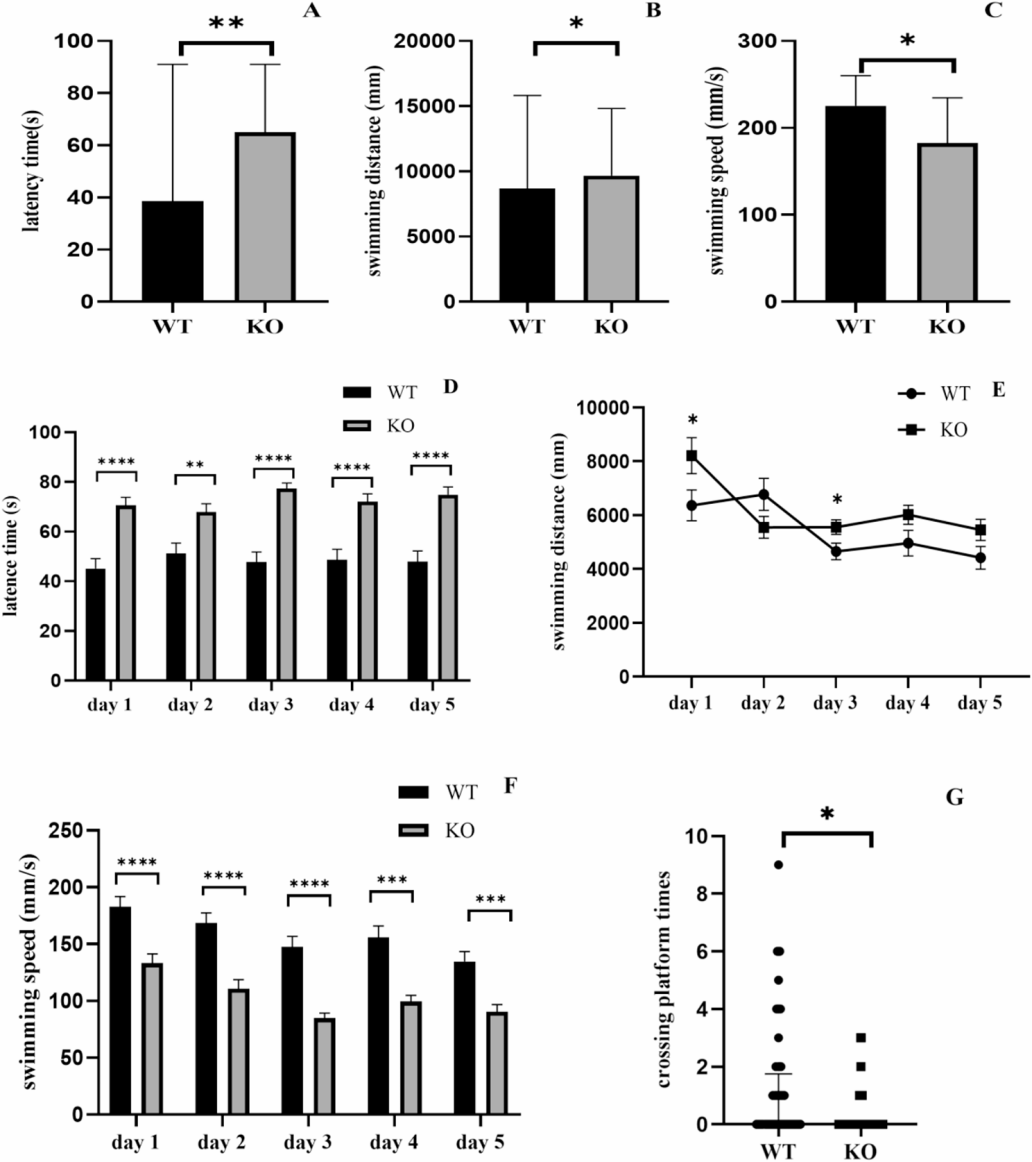
Morris water maze. (**A**, **B**, **C**) Escape latency time and mean swimming velocity in the cued learning test (KO mice: *n* = 25; WT mice: *n* = 20). Data are presented as the median with an interquartile range. *P < 0.05. The Mann-Whitney U test was used to compare two groups of data. (**D**, **E**, **F**) Escape latency time, total distance and mean swim velocity in the navigation test (KO mice: *n* = 25; WT mice: *n* = 20). Data are presented as the mean ± SD. *P < 0.05, **P < 0.01, ***P < 0.001, ****P < 0.0001. Unpaired t-tests were used to compare two groups of data. (**G**) Crossing platform times in the probe test (KO mice: *n* = 25; WT mice: *n* = 20). Data are presented as the median with an interquartile range. *P < 0.05. The Mann-Whitney U test was used to compare two groups of data

To evaluate the learning and memory ability of *DNAJC12* knockout (*DNAJC12* -/-) mice, a Morris water maze test was conducted, using 20 wild-type (WT) mice and 25 *DNAJC12* -/- mice aged 6–8 weeks. On the first day (Fig. 4A, B and C), the cued learning test was conducted. *DNAJC12*-/- mice showed significantly increased escape latency and lower mean swimming velocity compared to WT mice (*P* < 0.05). On days 2–6(Fig. 4D, E and F), the navigation test was conducted. The escape latency and total distance of all group mice decreased progressively over 5 days of training, regardless of the genotypes. However, *DNAJC12*-/- mice showed significantly longer escape latency time and slower mean swim velocity than WT mice (*P* < 0.05). Additionally, *DNAJC12*-/- mice traveled significantly longer distances on day 1 and day 3 (*P* < 0.05). On day 7, in the probe test (Fig. 4G), *DNAJC12*-/- mice showed significantly fewer platform crossings and longer distances in escape latency time in the platform zone compared to WT mice (*P* < 0.05).

### PAH, TH and TPH2 expression in DNAJC12-/- and DNAJC12+/+ mice groups

**Fig. 5:**
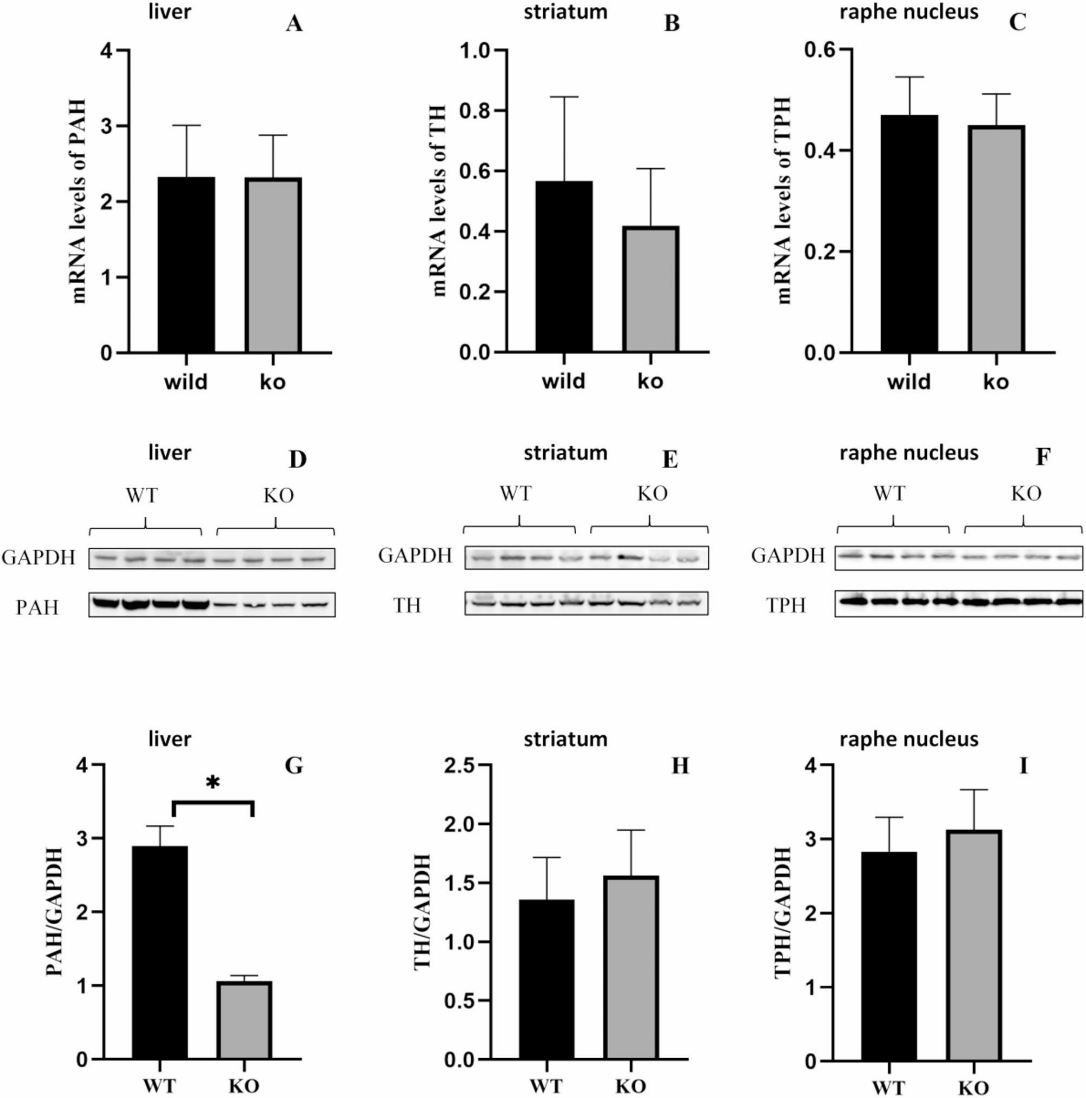
AAAHs expressions in RT-PCR and WB. (**A**, **B**, **C**) PAH, TH, and TPH2 mRNA expression, respectively in liver tissues, striatum tissues, and raphe nucleus tissues (KO mice: *n* = 8; WT mice: *n* = 8). (**D**, **G**) PAH, (**E**, **F**, **H**, **I**) TH, and TPH2 protein expression, respectively in liver, striatum, and raphe nucleus tissues (KO mice: *n* = 10; WT mice: *n* = 9). Data are presented as the mean ± SD. *P < 0.05. Unpaired t-tests were used to compare two groups of data

The target mRNA was detected by RT-PCR and analyzed by relative quantitative methods. There were no significant differences in the level of *PAH*, *TH*, and *TPH2* mRNA expression between the two groups (*P* > 0.05).The details as Fig. 5. The mice in the *DNAJC12* -/- group exhibited less PAH protein expression than those in the *DNAJC12*+/+ group (*P* < 0.05). There were no significant differences in TH and TPH2 between the two groups (*P* > 0.05). The details as Fig. 5.

### Brain monoamines, serum phe, and Tyr concentration

**Fig. 6:**
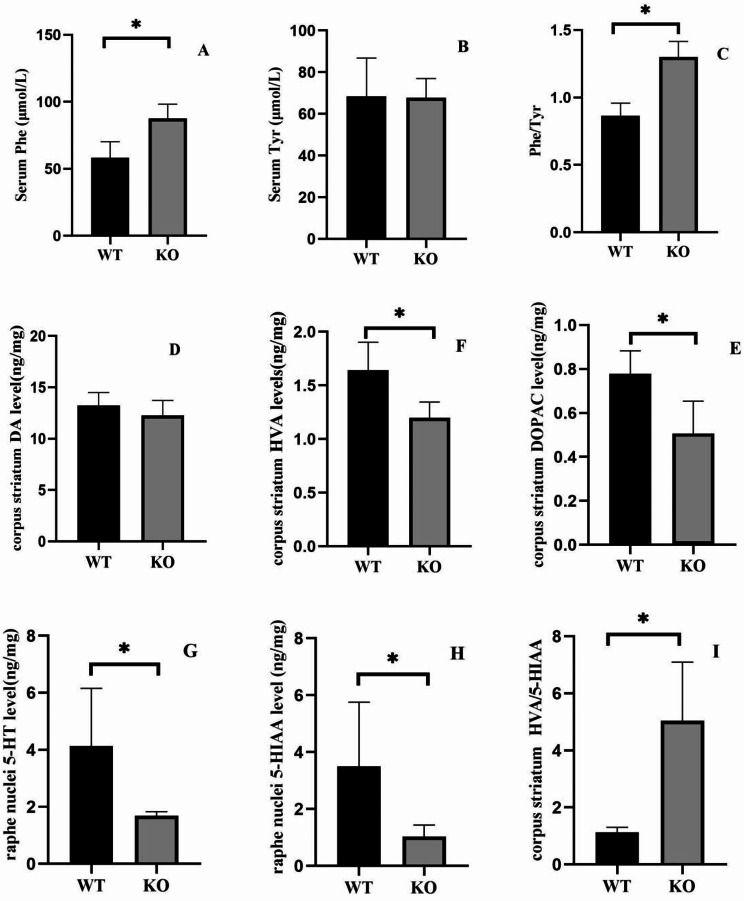
Serum Phe and Tyr and brain monoamines in WT mice and KO mice. (**A**, **B**) Serum Phe and Tyr concentration (KO mice: *n* = 5; WT mice: *n* = 5). (**C**) Serum Phe/Tyr ratio (KO mice: *n* = 5; WT mice: *n* = 5). (**D**) Brain DA, (**E**) DOPAC, (**F**) HVA, (**G**) 5-HT, (**H**) 5-HIAA, and (**I**) HVA/5-HIAA in the brain (KO mice: *n* = 5; WT mice: *n* = 5). Data are presented as the mean ± SD. *P < 0.05. Unpaired t-tests were used to compare two groups of data

DA in the *DNAJC12*-/- group was not significantly lower than in the WT group (*P* > 0.05). However, DOPAC and HVA levels were significantly lower in the *DNAJC12* -/-group than in the WT group (*P* < 0.05). Similarly, 5-HT and 5-HIAA were significantly lower in the *DNAJC12* -/- group than in the WT group(*P* < 0.05). The HVA/5-HIAA ratio was higher in the *DNAJC12*-/- group than in the WT group (*P* < 0.05). Additionally, serum Phe concentration and the Phe/Tyr ratio were significantly higher in the *DNAJC12*-/- group than in the WT group (*P* > 0.05). Tyr concentration in the *DNAJC12*-/- group showed no significant difference compared to the WT group (*P* > 0.05). The details are as Fig. 6.

## Discussion

Previous studies on *DNAJC12* mutations have primarily relied on in vitro cellular experiments, with a notable absence of in vivo models, limiting advancements in understanding the disease mechanisms. To bridge this knowledge gap, we have established the first *DNAJC12* knockout mouse model, providing a crucial tool for in vivo studies. Though the model does not exhibit all the clinical symptoms observed in human patients, such as muscle weakness, dystonia, convulsions, or Parkinsonism, it does present key features of the human phenotype, including impaired cognitive function, elevated phenylalanine levels, and decreased neurotransmitter levels, which are consistent with the clinical observations in patients. Additionally, we documented two patients with biallelic mutations in the *DNAJC12* gene, presenting with a disturbance in neurotransmitter levels. Building upon existing clinical and experimental research, our hypothesis posits that *DNAJC12* mutations result in diminished activity of aromatic amino hydroxylases, leading to hyperphenylalaninemia and reduced neurotransmitter levels, which consequently contribute to intellectual disability. This hypothesis has been substantiated through our research.

Both patients were identified through newborn screening due to elevated Phe concentrations. A definitive diagnosis was established through genetic testing and additional assessments. Clinical phenotype data were available only for Case 1, who presented with nocturnal eye closure, crying, and arching back in a reverse tension posture. These symptoms improved significantly with treatment using sapropterin dihydrochloride, L-dopa, and 5-hydroxytryptophan. These responses suggest that the symptoms may be due to a deficiency in monoamine neurotransmitters, a hypothesis further corroborated by subsequent animal experiments. Additionally, we observed his yellow hair color, a phenotype previously common in PKU patients but unreported in children with mutations in the *DNAJC12* gene. This observation may be linked to the disruption of tyrosine metabolism to melanin. Melanins serve as natural pigments in the skin, hair, and eyes. Melanogenesis, a complex process, commences with the conversion of the amino acid L-tyrosine to DOPA quinone. The subsequent addition of cysteine or glutathione to DOPA quinone initiates the formation of intermediates, which undergo further transformations and polymerization to yield the final product, pheomelanin. We posit that this symptom indicates a potential blockage in melanogenesis whereas it is not observed in the *DNAJC12* knockout mouse model. Whether changes in hair color are characteristic of the disease requires more case observations and further mechanistic studies.

Based on previous case reports and the findings from this study, the clinical symptoms associated with *DNAJC12* gene mutations show similarities to other forms of hyperphenylalaninemia. Patients with *DNAJC12* gene mutations also presented elevated blood phenylalanine concentrations, and untreated patients may have intellectual problems, muscle dystonia abnormalities, and psychiatric-behavioral problems. Similar to BH4 deficiency, BH4 loading assays or BH4 treatment in patients with *DNAJC12* gene are generally effective. Treatment with sapropterin and neurotransmitter supplementation can be effective in improving patient symptoms. However, there are also some differences between patients with *DNAJC12* gene mutations and those with other types of hyperphenylalaninemia. One major distinction is that the serum Phe levels in *DNAJC12* mutation patients usually remain no higher than 600µmol/L, whereas levels in classical PKU patients typically exceed 1200 µmol/L. Furthermore, while *DNAJC12* mutation patients often experience a Phe reduction of more than 30% during BH4 loading tests, their Phe levels do not consistently normalize [3, 4, 9, 16]. This response differs from BH4 deficiency but resembles BH4-responsive PKU. These findings suggest that DNAJC12 plays a role similar to BH4 in supporting PAH function, but it is not a subtype of BH4 deficiency. Additionally, MRI findings in patients with *DNAJC12* mutations are less pronounced than those seen in PKU patients. In previous reports, seven patients underwent MRI testing. Only a 13-year-old Chinese patient showed widened brain spaces, while the other patients, including three adults over 58 years old with intellectual disability and Parkinsonism, showed no abnormalities [2, 6, 17].

To further investigate the mechanism through which mutations in the *DNAJC12* gene lead to associated symptoms, we developed a *DNAJC12* gene knockout mouse model and conducted additional experiments. Cranial MRI scans were performed on both groups of mice (*n* = 3 per group, aged 8–10 weeks) using a 7T MRI instrument (data not shown). No significant abnormalities were detected in the model mice, consistent with observations in most human patients, who also showed normal MRI findings. However, we did not perform MRI scans at older ages, so it remains unclear whether the mice would exhibit abnormal MRI findings as they age. Since its inception in early 1981, the Morris Water Maze has been a pivotal tool in studying the brain mechanisms underlying learning and memory, rapidly becoming the classical method for assessing these abilities in mice [18]. Our findings indicate that *DNAJC12* knockout mice exhibit impaired learning and memory abilities, aligning with cognitive impairments observed in clinical patients. Traditionally, DNAJC12 has been regarded as a chaperone for AAAHs, with mutations in the *DNAJC12* gene leading to reduced AAAHs activity and consequently, cognitive deficits. Research by Huttlin EL et al. and Anikster et al. has demonstrated DNAJC12 interactions with PAH, TH, and TPH2^2, 15^. Furthermore, Gallego et al. observed that *DNAJC12* gene variants might impact PAH and TH protein expression, but not TPH [7]. Consequently, we initially posited that DNAJC12 serves as a chaperone for all AAAHs. However, our results reveal a reduction in PAH expression, while TH and TPH levels remain unchanged. In the knockout group, PAH protein expression was observed to be diminished, whereas mRNA expression remained unaffected. This suggests that *DNAJC12* gene mutations regulate PAH at the post-transcriptional processing level. Members of the DNAJ/HSP40 family function as chaperones, regulating the HSPA/HSP70 and HSPC/HSP90 families. DNAJ proteins, through their highly conserved J domain, interact with HSP70 to ensure the homeostasis of substrate proteins, facilitating proper folding and degradation. Mutation in the *DNAJC12* gene might lead to improper folding of PAH, resulting in the degradation of structurally abnormal proteins and, consequently, reduced PAH expression. Furthermore, BH4 serves as a cofactor for the AAAHs family, aiding in their correct folding. The positive response to the BH4 loading test in nearly all patients with *DNAJC12* gene mutations suggests a functional similarity between DNAJC12 and BH4^2–4^.

To further investigate the impact of DNAJC12 on PAH-related pathways, we analyzed phenylalanine and tyrosine concentrations in the blood of *DNAJC12* knockout mice. The elevation of blood Phe and Phe/tyr was statistically significant, but the degree of elevation was minor. Given that PAH catalyzes the conversion of Phe to Tyr, these results may imply a partial blockade in this conversion process, without causing a substantial elevation in Phe levels. In patients with *DNAJC12* gene mutations, Phe levels are typically mildly elevated, aligning with our findings. The minor increase in Phe observed is intriguing, as it does not appear to correlate with significant clinical symptoms.

Although there was no significant decrease in protein expression of TH and TPH2 in the WB experiments, subsequent results reveal that *DNAJC12*-/- mice exhibit lower levels of their downstream product, DOPAC, HVA, 5-HT, and 5-HIAA, suggesting disruptions in DA and serotonin synthesis. Monoamine neurotransmitters are crucial for neuromodulation, impacting motor control, motivation, reward, cognitive function, and maternal and reproductive behaviors [19, 20]. Imbalances in monoamine neurotransmitter levels can contribute to bipolar disorder, addiction, hypertension, dystonia, attention-deficit/hyperactivity disorder, autism, and schizophrenia. In our case study, the patient exhibited symptoms including nocturnal eye closure, crying, and arching back with reverse tension. Remarkably, these symptoms resolved following the administration of oral Sapropterin dihydrochloride, L-dopa, and 5-hydroxytryptophan. This observation could explain the cognitive impairments and abnormal behaviors noted in *DNAJC12* knockout mice. The subsequent challenge lies in identifying the causes of reduced neurotransmitter levels. Traditionally, decreased monoamine neurotransmitters have been attributed to malfunctioning of tyrosine hydroxylase and tryptophan hydroxylase, the impaired conversion of phenylalanine to tyrosine, and disruptions in large neutral amino acid transport [21–23]. However, there were no significant differences in TH and TPH mRNA and protein expression levels between *DNAJC12* -/- mice and WT mice. we did not measure their activities to know whether DNAJC12 affects its enzyme activity through other pathways, leading to a decrease in downstream products. As for the large neutral amino acid competition doctrine, neither elevated phenylalanine nor decreased tyrosine in clinical patients and mouse models is sufficiently high to support a significant decline in pro-neurotransmitters. Beyond TH, other proteins like dopamine decarboxylase (DDC) are significant in DA metabolism. The precise mechanism underlying the decrease in monoamine neurotransmitters warrants further investigation.

### Limitations

There are several limitations in this study. Firstly, our assessment of cognitive function in the mouse model was solely based on the Morris water maze, which may not comprehensively capture the full spectrum of behavioral issues. Secondly, while we investigated the impact of *DNAJC12* gene knockout on the mRNA and protein expression of AAAHs, its effects on their folding and degradation have not been explored. Lastly, although we established that *DNAJC12* gene knockout leads to decreased PAH expression, the direct correlation between reduced PAH expression and impaired cognitive function remains unconfirmed. Future research will delve deeper into the pathogenic mechanisms of *DNAJC12* gene knockout to address these limitations.

## Conclusions

We report two patients with four novel mutations within the *DNAJC12* gene (c.158-1G > A, c.262 C > T, c.473 C > G, c.102delT) and have established the first *DNAJC12* gene knockout mouse model based on the patients’ mutations location. Both of the patients and the model mice present hyperphenylalaninemia and neurological symptoms. Further experiments revealed that knocking out the *DNAJC12* gene in mice leads to decreased PAH protein expression and reduced brain monoamine neurotransmitter levels. It may suggest that the secondary effects of the *DNAJC12* gene mutation involve regulating the expression of PAH protein, potentially occurring at the post-transcriptional level.

## Data Availability

Please contact the corresponding author for data requests.
